# E-Learning during COVID-19: perspectives and experiences of the faculty and students

**DOI:** 10.1186/s12909-022-03383-x

**Published:** 2022-04-28

**Authors:** Sisi Li, Chunhui Zhang, Qijun Liu, Kuang Tong

**Affiliations:** 1 Center for Teaching and Learning Development, Southern Medical University, Shatai Road 1023, Baiyun District, Guangzhou, 510515 Guangdong China; 2 Office of Academic Affairs, Southern Medical University, Baiyun District, Shatai Road 1023, Guangzhou, 510515 Guangdong China

**Keywords:** E-learning, COVID-19, Perceived effectiveness, Medical university, Faculty, Medical student

## Abstract

**Background:**

Aimed to corroborate students' and faculty's experiences with e-learning during the current pandemic.

**Methods:**

A cross-sectional study was conducted from February to June 2020. Seven surveys were distributed electronically to all undergraduate students and the faculty (4 to students and 3 to teachers) at the Southern Medical University (China). Descriptive statistics and t-tests were used to analyze the data. Statistical significance was set at *p* < .05.

**Results:**

Most students had some exposure to e-learning prior to the all e-learning regiment, contrasted with close to 90% of teachers having no or very limited experience. Students' perceptions of the most helpful e-learning activities did not change significantly overall (Week 3 vs. Week 9). Approaching 60% of students (Week 9) did find online discussion/Q&A/forum helpful, an increase from less than 30% (Week 3). Among teachers, gaps emerged (Week 9) between e-teaching activities used and their perceived effectiveness. Despite pre-recorded lectures being the most frequently used method, the least gap was associated with live-stream lectures—the least used. Over time, teacher's perceived effectiveness of e-teaching vs. in-person teaching did not differ significantly overall. When the results among students (Week 7) and teachers (Week 9) were corroborated, a slightly higher percentage of teachers viewed online teaching to be less effective than in-person teaching and a slightly higher percentage of teachers viewed online teaching as far less effective. For preferred learning modes after the resumption of in-person learning, students' preferences did not differ significantly overall (Week 3 vs. week 9). Surveys conducted in Week 9 found that a slightly higher percentage of students (~ 70%) than teachers (~ 60%) preferred some forms of hybrid learning and a lower percentage of students preferred face-to-face learning only. Approximately three quarters of teachers responded that at least 50% of course materials could be mastered by students on their own.

**Conclusions:**

Overall, the perceived effectiveness of e-learning among students and teachers has not changed significantly over time. Nor have students' preferences shifted significantly for various learning modes after the in-person learning resumed. However, informative directional trends have emerged. Our research illustrates empirically the need to corroborate students' and instructors' experiences over time to inform more holistic improvements of e-learning.

## Background

There have been a plethora of published reports on e-learning since the start of the COVID-19 pandemic, including a number of studies examining the experiences and perspectives of students and/or teachers at medical education institutes. For example, Li et al. [1] presented students’ initial perspectives on online learning during COVID-19, based on a large nationally-representative sample across 90 medical schools in China. The team found that the most common problems encountered by students were network congestion (77%), insufficient interactions between learners and instructors (45%), lack of timely feedback & answers to questions (10%), and poorly-prepared course materials by instructors (6%). Approximately 62% of students were satisfied or extremely satisfied with the e-learning programs during the pandemic. Motte-Signoret et al. [2] surveyed Year-5 medical students, pediatric residents, neonatal fellows, pediatrics instructors in France—146 learners and 26 instructors—for their perceptions of medical education conducted during COVID-19. More than half of the students (59%) and a slightly higher percentage of teachers (69%) responded that they did not receive quality trainings as expected. Elshami et al. [3] examined the satisfaction with online learning during the current pandemic among 358 students and 70 teachers in UAE. Approximately 72% of students had no prior experience with online learning, slightly less than 82% among teachers. Approximately 69% of students were less satisfied with online learning and 42% would not recommend it. A higher percentage of teachers (63%) was more satisfied with online than in-person learning, and 92% of them also reported that students demonstrated higher enthusiasm toward online than traditional learning. Schlenz et al. [4] surveyed 242 dental students and 35 lecturers in Germany for their perspectives on the implementation of online learning due to COVID-19 and found that students would like to see around 50% of the theoretical portion of the course delivered online, while teachers would like less than 40%. Kim et al. [5] surveyed 318 Year-1 to Year-4 medical students and 44 teachers in South Korea for their experiences with the medical education delivered during COVID-19, and found that students were generally satisfied with the courses (3.97/5 based on a 5-point scale) and that teachers' satisfaction with the guide for online lectures, overall process of online class operation, and technical aspects of online lecturers ranged from 3.77 to 4.05. These studies contributed valuable learnings to the current understanding in the field of e-learning. At the same time, they recruited relatively small samples of teachers and presented a snapshot of the experiences of students and/or teachers at a particular time point, instead of a longitudinal view.

In response to the global outbreak of COVID-19, the Ministry of Education of the People's Republic of China instituted a policy of "Disrupted Classes, Undisrupted Learning". This policy mandated all schools in China to implement remote learning and teaching by delivering all curricula online.

Therefore, to expand the scopes of previous studies and bridge gaps in the current literature, a study was undertaken to investigate the experiences of students and teachers with the all e-learning regimen implemented at a local medical university in China. The study aimed to:

1. Recruit larger samples of students and teachers and corroborate, in the same study, their perspectives on the e-learning mandated during the current pandemic; and
2. Examine how their experiences and perspectives evolved over time.

Empirically-derived evidence could then be gleaned from the findings to facilitate more holistic improvements of e-learning in the future.

## Methods

### Study participants

There are two types of medical schools in China: an independently operated medical college, and a school as part of a university. A broad range of disciplines are taught at both types of schools, such as clinical medicine, Chinese medicine, basic medicine, public health, pharmacy, nursing, and health management. The Southern Medical University (SMU) is an independent medical school located in southern China, comprising 15 Schools spanning diverse disciplinary domains and a faculty size of about 1600. The SMU enrolls approximately 3200 undergraduate and 2100 graduate students each year.

### Instrument

As part of this cross-sectional study, 4 questionnaires for students and 3 for teachers were developed by 3 coauthors of this report, based on an extensive literature review. One worked at the Teaching and Learning Center of the SMU, specializing in educational technology, and the other 2, at the Office of Academic Affairs with a specialty in Clinical Medicine. The draft surveys were presented to 6 faculty members affiliated with different medical schools outside the SMU, whose specialties ranged from basic science, public health, nursing, to clinical medicine. Ensuing discussions were convened between the researchers developing the surveys and the 6 faculty members to ensure face and content validity of the surveys.

As each round of survey results from students became available, they were shared with the faculty members so they could adjust their teaching as they saw needed. Similarly, the survey results from teachers were shared with the Center for Teaching and Learning Development, so the Center could prepare faculty development activities tailored to teachers' needs. From Week 5 through Week 8 during this study, a series of online trainings were held for teachers (1–2 h per day & 3–5 days per week), including, for example, how to use the online platform and how to prepare videos. Faculty members also shared, during these trainings, their first-hand experiences, tips, best practices, and success stories.

The objectives and topics of interest of each survey are presented in Tables 1 and 2, where student surveys are labeled as "S" and faculty surveys, as "F".

**Table 1:** Objectives of student surveys

Survey #	Time	Objective
S1	Week 1	Students' prior experiences with e-learning (platforms), e-learning preferences & habits, and preferences of different learning modes during COVID-19
S2	Week 3	Students' e-learning habits & preferences, perceived effectiveness of different e-learning activities, preferences of different learning modes after the resumption of in-person learning
S3	Week 7	Students' e-learning habits & preferences, perceived effectiveness of different e-learning activities & different learning modes, e-learning challenges encountered
S4	Week 9	Students' e-learning habits & preferences, perceived effectiveness of e-learning (by disciplinary domain) & different e-learning activities, preferences of different learning modes after the resumption of in-person learning

**Table 2:** Objectives of faculty surveys^a^

Survey #	Time	Objective
F1	Week 1	Instructors' prior experiences with e-teaching, e-teaching support received & expected, preferences of e-teaching activities, methods to gauge students' engagement (with e-teaching), satisfaction with current e-teaching outcomes, perceived effectiveness of different learning modes
F2	Week 3	Instructors' levels of comfort with current e-teaching, e-teaching habits, satisfaction with current e-teaching outcomes, perceived effectiveness of different learning modes, challenges encountered, interactions with students during e-teaching, methods to gauge students' engagement (with e-teaching), e-teaching benefits experienced, types of support needed
F3	Week 9	Instructors' prior concerns about e-teaching, helpful types of support received, sources of e-teaching materials, e-teaching habits, e-teaching activities utilized, perceived effectiveness of e-teaching activities & different learning modes, challenges encountered, preferences of different learning modes after the resumption of in-person teaching

^a^There was no survey conducted among teachers in Week 7, as they were attending a series of online trainings designed to support them

### Procedure

From February to June 2020, questionnaires were distributed electronically to all the faculty members and undergraduate students who have registered for courses. Participation in the surveys was voluntary and anonymous. The questionnaire was self-administered and did not contain identifying information of respondents. Questionnaires which were not filled out completely were excluded from subsequent analyses.

### Data analysis

Descriptive statistics was used to summarize participants’ characteristics and responses, and categorical variables were presented using frequencies and percentages. Where applicable, t-tests were conducted where *p* < 0.05 was deemed statistically significant. All statistical analyses were performed using IBM SPSS version 26.0.

## Results

### Characteristics of respondents

The distributions of gender, grade year, and academic major were comparable across the 4 student surveys (Table 3). Students at the SMU start their clinical clerkship in the 2^nd^ semester of their 4^th^ year of study and attend clinical rotations at participating hospitals starting in their 5^th^ year of study.

**Table 3:** Characteristics of student respondents

	% of Total Respondents
Grade year	
1^st^-year	30
2^nd^-year	30
3^rd^-year	25
4^th^-year	15
5^th^-year	15
Gender	
Female	65
Male	35
Academic major	
Clinical medicine	35
Health management	35
Chinese medicine	15
Public health	10
Pharmacy	6
Nursing	6
Laboratory medicine & biotechnology	4
Basic medicine	4
Forensic medicine	1.5

The distributions of gender, age, professional credential, and academic department were also comparable across the 3 faculty surveys (Table 4).

**Table 4:** Characteristics of faculty respondents

	% of Total Respondents
Age (years)	
Younger than 35	20
35 ~ 45	50
46 ~ 55	25
Older than 55	5
Gender	
Female	55
Male	45
Academic position	
Assistant professor	40
Associate professor	40
Professor	20
Academic department	
Clinical^a^	60
Non-clinical^b^	40

^a^Including clinical medicine, Chinese medicine, and nursing

^b^Including basic medicine, public health, pharmacy, laboratory medicine and biotechnology, and health management

The number of respondents and return rate for each survey are presented in Table 5:

**Table 5:** Respondents and return rates^a^

Time of Survey	Student		Faculty	
	No. of Respondents	Return Rate	No. of Respondents	Return Rate
Week 1	4387	49%	415	66%
Week 3	5675	63%	357	57%
Week 7	4640	51%	NA	NA
Week 9	6115	68%	158	25%^b^

^a^The return rate has been rounded up or down to the nearest integer

^b^At the SMU, each semester is also punctuated by sessions which do not necessarily last the entire semester. Hence, this lower rate of return may be the function of fewer teachers around to participate in the survey as certain sessions were already concluded by the time of the survey

### Baseline: the e-learning experiences and perspectives of students and faculty

Students had had various degrees of exposure to e-learning prior to the mandated all e-learning regimen initiated by the SMU. According to the first student survey conducted in Week 1, 73% of respondents had experience with the MOOC (Massive Open Online Course) created by the China University, 32% with Treenity produced by the Able Company, and 26% with Moodle designed by the SMU.

During the same survey, students were asked about their views of various learning modes. Approximately 55% of them responded that "most learning should be done in person, with the rest by e-learning", 37% responded that "most learning can be done online, with the rest in person", and 9% responded that "all learning can be done online".

During the survey among the faculty members also conducted in Week 1, 60% of them responded that they had had no experience with e-teaching, 26% having used some forms of e-teaching, 11% employing e-teaching for at least 2 semesters, and 3% adopting e-teaching for only 1 semester.

### E-learning activities used by students and teachers and their perceived effectiveness

**Fig. 1:**
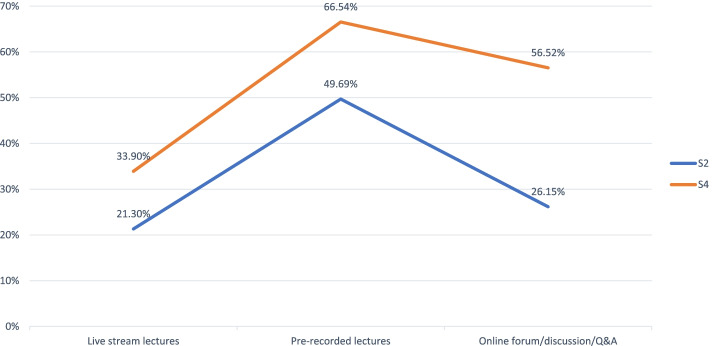
Selected e-learning activities used by students and perceived to be effective (multiple-choice)^*^. ^*^Based on responses to Q11(S2, week 3) and Q11 (S4, week 9)

When asked which learning activities were deemed the most helpful, students' responses did not differ significantly overall between Survey 2 (Week 3) and Survey 4 (Week 9) (Table 6). However, there appeared a directional trend suggesting that, over time, increasing percentage of students found certain e-learning activities helpful for their learning. In particular, approaching 60% of students in Survey 4 (Week 9) found online discussion/Q&A/forum helpful for their learning, an increase from less than 30% during Survey 2 (Week 3) (Fig. 1).

**Table 6:** Which of the following activities are most helpful for your learning (multiple-choice)?^a^

	S2	S4
Review course content before class	1720	1208
Participate in live-stream lectures	1209	2073
Use pre-recorded lectures	2820	4069
Participate in online Q&A/discussions/forums	1484	3456
Participate in in-class tests/examinations	1619	2428
Complete assignments	1748	2037
Others (please specify)	48	34
Total (Responses)	10,648	15,305
Total (Respondents)	5675	6115
*t*(statistic)	0.286	
*df*	12	
*t*(critical value), *p* = 0.05	2.179	

^a^Based on responses to Q11 in Student Survey 2 (S2, Week 3), Q15 in Student Survey 3 (S3, Week 7), and Q11 in Student Survey 4 (S4, Week 9)

**Fig. 2:**
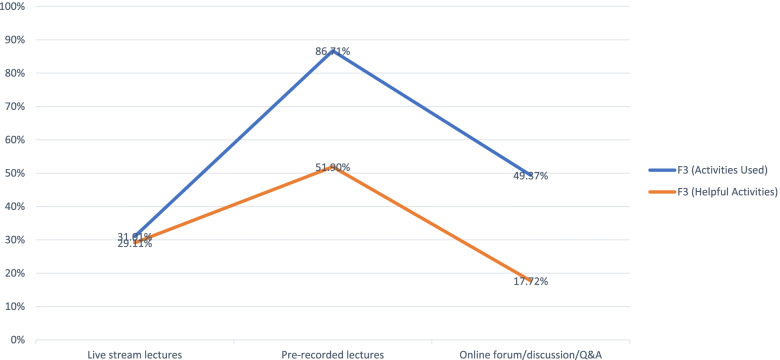
Selected e-teaching activities used by teachers & their perceived effectiveness (multiple-choice). ^*^Based on responses to Q17 and Q19 in Faculty Survey 3 (F3, Week 9)

During Survey 3 (Week 9) among teachers, gaps emerged between selected e-teaching activities engaged and teachers' perceived effectiveness of these activities. Interestingly, the largest gap was associated with pre-recorded lectures—the most frequently used method, while the least gap was associated with live-stream lectures—the least common method used (Fig. 2).

### E-learning vs. in-person learning: perspectives of students and the faculty

**Fig. 3:**
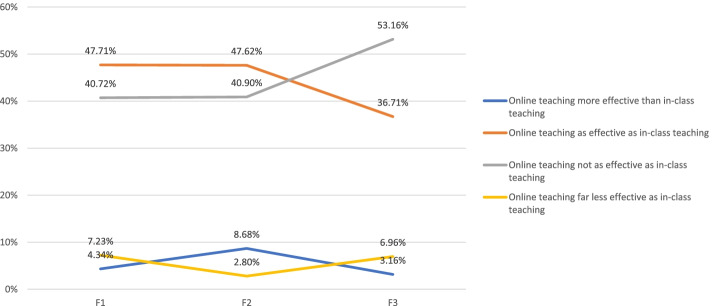
Evolution of faculty members' perceived effectiveness of online teaching vs. in-person teaching (single-choice)^*^. ^*^Based on responses to Q8 (F1, Week 1), Q8 (F2, Week 3), and Q24 (F3, Week 9)

Teacher's perceived effectiveness of online teaching versus in-person teaching did not differ significantly overall over time (Table 7). However, there appears a directional trend suggesting that an increasing percentage of teachers viewed on-line teaching not to be as effective as in-person teaching, that a decreasing percentage of teachers felt that online teaching was as effective as in-person teaching, and that an increasing percentage of teachers perceived online teaching to be far less effective than in-person teaching (Fig. 3).

**Table 7:** Can online teaching be as effective as in-class teaching (single-choice)?^a^

	F1	F3
Online teaching more effective than in-class teaching	18	5
Online teaching as effective as in-class teaching	198	58
Online teaching not as effective as in-class teaching	169	84
Online teaching far less effective as in-class teaching	30	11
Total (Respondents = Responses)	415	158
*t*(statistic)	0.248	
*df*	6	
*t*(critical value), *p* = 0.05	2.447	

^a^Based on responses to Q8 (F1, week 1) and Q24 (F3, week 9)

**Fig. 4:**
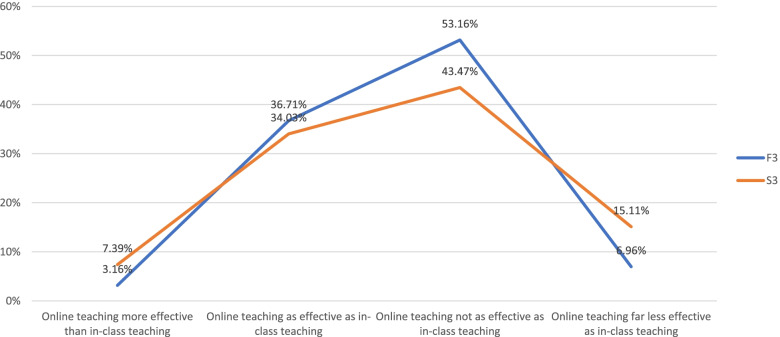
Perceived effectiveness of online teaching vs. in-person teaching: faculty members vs. students^*^. ^*^Based on responses to Q24 (F3, week 9) and Q18 (S3, week 7) (single-choice); *t* = .987, *t*(critical value) = 2.447 (*df* = 6, *p* = .05)

When corroborating students' (S3, Week 7) and teachers' feedback (F3, Week 9), we found no statistically significant difference between the two groups (*t* = 0.987; *t* critical value = 2.447, *df* = 6, *p* = 0.05). However, we observed a *directional trend* that a slightly higher percentage of teachers than students felt that online teaching was not as effective as in-person teaching (Fig. 4).

### Preferred learning modes after the resumption of in-person learning

**Fig. 5:**
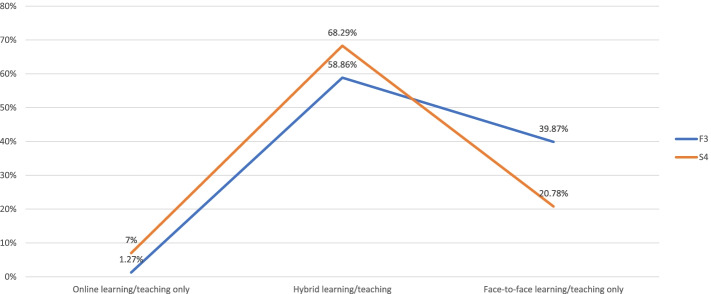
After the resumption of in-person learning, what type of learning will you be more willing to engage in (single-choice)?^*^. ^*^Based on responses to Q14 (S4, Week 9) and Q25 (F3, Week 9); *t* = .960, *t*(critical value) = 2.776 (*df* = 4, *p* = .05)

**Fig. 6:**
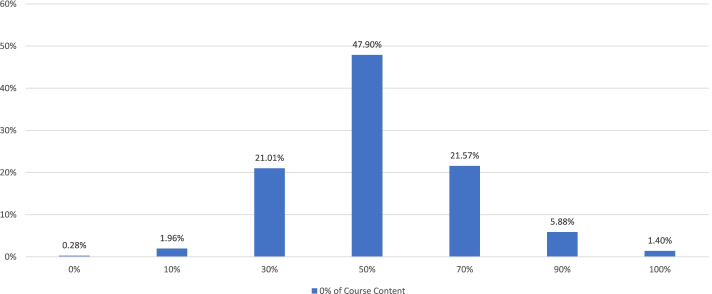
Based on your experience with e-teaching during COVID-19, how much of the course content you feel can be mastered by students on their own (single-choice)?^*^. ^*^Based on responses to Q17 (F2, Week 3). For the category of "0% of Course Content", the percentage was rounded down from 0.28%

Students' preferences of various learning modes after the resumption of in-person learning did not change significantly overall between Survey 2 (Week 3) and Survey 4 (week 9) (Table 8). When we compared students' with teachers' responses collected during the 9^th^ week of the survey (S4 and F3, respectively), we found no statistically significant difference between the two groups (*t* = 0.960; *t*(critical value) = 2.776, *df* = 4, *p* = 0.05). However, a directional trend showed that a slightly higher percentage of students than teachers preferred some forms of hybrid learning and a lower percentage of students preferred face-to-face learning only (Fig. 5). Meanwhile, students' initial enthusiasm over various forms of blended learning appeared to have tapered somewhat over time, dropping from higher than 90% of responses (S1, Week 1) to approximately 70% (S4, Week 9) (as reported earlier under "Baseline" in this paper). On the other hand, when asked how much of the course content could be mastered by students on their own, approximately three quarters of teachers responded at least 50% (Fig. 6).

**Table 8:** After the resumption of in-person learning, what type of learning will you be more willing to engage in (single-choice)?^a^

	S2	S4
E-learning only	273	428
Hybrid-learning with e-learning as the mainstay and in-person learning as a supplement	1289	1555
Hybrid-learning with in-person learning as the mainstay and e-learning as a supplement	2408	2615
Face-to-face learning only	1517	1271
Not sure	188	246
*Total (Respondents = Responses)*	5675	6115
*t(statistic)*	0.886	
*df*	8	
*t*(critical value), *p* = 0.05	2.306	

^a^Based on responses to Q15 (S2, Week 3) and Q14 (S4, Week 9)

## Discussions

Overall, the perceived effectiveness of e-learning among students and teachers did not change significantly during the survey periods (Tables 6 and 7). Nor did teachers' perspectives shift significantly in terms of their preferences for various learning modes after the resumption of in-person teaching (Table 8). These results are not entirely surprising, given that the series of surveys conducted spanned approximately 8 weeks—a relatively short time frame for major shifts in learning preferences or behaviors to take place. However, a number of informative directional trends did emerge from the surveys:

### Utilization and perceived effectiveness of e-learning/e-teaching among students and teachers

As shown in Figs. 1 and 2, pre-recorded lectures were the most frequently used e-learning functionality by both students and teachers. However, the most noticeable increase in adoption over time among students occurred with online forum/discussion/Q&A. Similarly, the least gap between the actual use and perceived effectiveness among teachers appeared to be associated with live-stream lectures. Both live-stream programs and online discussions afford students and teachers more real-time interactions, and the lack of interactions has been consistently cited as one of the disadvantages associated with e-learning [1, 2, 5]. The benefits of student-faculty and student-student communications have been documented in the literature [6–8] Thus, the above trends emerging from the current research lend further support to the need to direct resources and trainings to increase teachers' proficiency in utilizing these real-time tools. In the meantime, although virtual interactions cannot replicate all the "soft" features of face-to-face interactions, they do usher in new possibilities as mediated by technology which can transcend the constraints of space (e.g. geographic location) and time (e.g. frequency). These new possibilities were also keenly observed by Su et al. who reviewed hundreds of local publications on the implementation, challenges, and perspectives of virtual classrooms for medical education during COVID-19 and beyond. The publications reviewed were drawn from 2 main local databases: China National Knowledge Infrastructure (CNKI) and The VIP Chinese Journal Platform [9].

Additionally, for the perceived effectiveness of e-learning versus in-person learning, Fig. 4 stacks students' feedback against teachers' as collected from the last survey in Week 9. The graph shows that a slightly higher percentage of teachers viewed online teaching as less effective. This somewhat divergent view between students and teachers may reflect divergent degrees of exposure to e-learning between the two groups prior to the installment of the all e-learning program at the SMU. As documented earlier in this report, close to three quarters of student respondents had experience with MOOCs and a third as well as a quarter, respectively, had used other e-learning platforms. Contrariwise, close to 90% of faculty respondents had little or very limited exposure to e-teaching. This finding confirms the observations presented by Wang et al. that students' prior learning experiences were positively correlated with their evaluation of the online education and their satisfaction [10].

### Favorable nod to hybrid learning (various combinations) among students and teachers

When asked which learning mode they preferred after the in-person learning/e-teaching resumed, approximately 60% of teachers and 70% of students cast their votes for some forms of hybrid learning. Furthermore, approximately three quarters of teachers responded that at least 50% of the course content could be mastered by students on their own. These rates are higher than some observations reported in the literature. For example, Motte-Signoret et al. surveyed Year-5 medical students, pediatric residents, neonatal fellows, and pediatric instructors in France and reported that 28% learners and 38.5% teachers responded that online teaching should continue after COVID-19 [2]. Schlenz et al. surveyed dental students and instructors in Germany and reported that students demanded 53.2% of theoretical curriculum to be offered online, contrasted with 38.6% among instructors [4]. Kim et al. surveyed medical students and teachers in Korea and reported that 84% of students would like to maintain online programs after COVID-19, contrasted with 48% among teachers [5].

We think a number of factors might have contributed to our findings. First, students' initial enthusiasm over e-learning did taper somewhat during the survey periods, possibly as resulted from the time needed for less experienced teachers (with e-teaching) to ramp up their learning. Hence, it stands to reason that teachers' comfort level and fluency with e-teaching at least partially contributed to students' tapering enthusiasm over hybrid learning. Nonetheless, most students' prior exposure to e-learning appeared to have sustained their overall favorable view of e-learning. For teachers, the extra trainings which they received from Week 5 through Week 8 (based on the feedback collected during earlier rounds of surveys) also assisted them with much-needed support as they navigated through their learning curves. No less importantly, prior to the emergency switch from all face-to-face learning to all online learning, e-learning had not been a learning mode that was integral to the medical education at the SMU. Paradoxical as it may seem, the emergency created by the current pandemic essentially "forced" students and teachers alike to seriously consider virtual learning as a viable option that could not only rival in-person learning in some cases but surpass the latter in others.

Hybrid learning not only affords the educational institutes and faculty the logistical flexibility, but can also yield tangible pedagogical benefits by integrating student's independent and self-paced learning (aided by e-learning) with in-person classroom learning where emphasis is placed on richer exchange of face-to-face interactions. Blending passive and active learning through the implementation of flipped classrooms, for example, can be a fruitful design to deliver such optimized learning.

After in-person learning was resumed in May 2020, the SMU rolled out a new policy on hybrid-learning which mandates that at least 20% of suitable course materials are to be delivered through virtual self-learning by students themselves. In terms of assessing the learning outcomes, students' performance in the final examination cannot exceed 50% of their total score for a particular course, with the rest of the outcome assessment being anchored in such activities as in-video quiz, peer review, case study, and student presentation conducted in class and online. This policy was created based on the feedback collected from the 7 surveys conducted and reported in this paper. A qualitative research based on interviews with policy-makers, students, and teachers are also ongoing to study the effects of this new policy.

For policy-makers and administrators, the best practices benchmarked by Jiang et al. offer additional worthy fruit for thought, who surveyed how online teaching was conducted to educate medical students in China during COVID-19. For example, a "responsive educational system" can be created to encourage early adopters to become leaders in the community to promulgate the "multiplier effect". Existing quality online teaching resources can be identified so not all teaching/learning materials need to be built from scratch [11].

### Broad-based measures for e-learning outcome assessment

As in the face-to-face environment, assessment is instrumental in facilitating continuous improvements in active learning in the online environment [12]. Based on responses to Question 10 (multiple-choice) in Survey 3 (Week 9), teachers reported a number of methods employed to assess students' learning outcomes and engagement in e-learning, for instance, in-video quiz (32%), assignment & test (66%), peer review (34%), taking attendance without a prior announcement (18%), sign-in (28%), and system-compiled statistics (48%). These methods echoed a myriad of assessment options presented in a systemic review by Wei et al. [13] who reviewed 65 peer-reviewed articles published from 2017 through 2019.

As stressed by Chen et al. [14], the methods used for evaluating the effectiveness of e-learning need to align with desired outcomes. As e-learning harbors real opportunities to expand students' skills and capabilities beyond knowledge acquisition—for instance, self-directed learning, time management, and team work, methods of assessment commensurate with the versatility of e-learning deserve serious considerations and should include innovative approaches beyond what have been practiced traditionally. The three-pronged model concluded by Wei et al.—assessing the cognitive (including knowledge and intellectual skills), behavioral, and affective outcomes—offered a holistic approach to not only capture the traditional measures but also expand the toolbox of e-learning/e-teaching assessment. For example, self-assessment, discussion forum, and writing project can be used to assess learners' intellectual skills; user data recorded by the platform can be analyzed to track the trajectory of learners' engagement; and learning analytics techniques and educational data mining can be implemented to extract user data to gain insights into learners’ behavioral outcomes. Vonderwell & Boboc [15] also documented formative assessment techniques used by two instructors in their respective online courses at the graduate level, such as the minute paper, hook questions, and discussion forum.

### Limitations

A number of limitations need to be heeded when extrapolating findings from the current study. First, the swift transition in a large scale from all face-to-face learning to all online learning necessitated by the sudden outbreak of the pandemic created a unique set of externalities that likely conditioned the experiences of students and faculty. Their e-learning experiences outside an emergency like a pandemic thus might not necessarily be in parallel with what emerged from the current study. Second, no major shifts in learning behaviors and preferences have been found among students and teachers, which might be partly attributable to the relatively short time horizon of our study (approximately 8 weeks). Therefore, follow-on research with longer time intervals between surveys and over a longer period of study time will be needed to confirm or challenge the trends distilled from our study. Third, the current study reported the aggregate experiences and perspectives of medical students at the SMU. These observations may not bear out among subgroups of students of different grade years (pre-clinical vs. clinical phase) and course types. The optimal ratio between on- and off-line learning in a hybrid model also likely varies with the learning stage of students. Additional cohort studies focusing on these subgroups will be needed to derive more complete insights of students' experiences with e-learning.

## Conclusions

Overall, the perceived effectiveness of e-learning among students and teachers have not changed significantly over time. Nor have students' preferences shifted significantly for various learning modes after the in-person teaching resumes. However, a number of informative directional trends have emerged. Pre-recorded lectures are the most frequently used e-learning functionality by both students and teachers. However, the most noticeable increase in adoption over time among students occurs with online forum/discussion/Q&A. Similarly, the least gap between the actual use and perceived effectiveness among teachers appears to be associated with live-stream lectures. Both live-stream programs and online discussions afford students and teachers more real-time interactions. When asked which learning mode they prefer after the in-person learning/teaching resumes, approximately 60% of teachers and 70% of students cast their votes for some forms of hybrid learning. Approximately three quarters of teachers respond that at least 50% of the course content can be mastered by students on their own. These rates are higher than some observations reported in the literature. Teachers employ a number of ways to assess students' learning outcomes and engagement in e-learning, such as in-video quiz, assignment & test, peer review, taking attendance without a prior announcement, sign-in, and system-compiled statistics. These methods echo a myriad of assessment options currently practiced in the field.

Both students and instructors are key stakeholders in optimizing learnings (e-learning included). Our study thus serves as an empirical example of how the experiences and perspectives of both can be corroborated to facilitate learning improvements more holistically. Contrary to the majority of extant studies based on snap-shot surveys, the current study is designed to monitor the evolution of students' and instructors' experiences and behavioral shifts over time, so empirical insights can be gleaned for policy-makers to instrument curricular adjustments and/or render support (including allocating resources required) in a more timely manner. Furthermore, our study illustrates that valuable learnings can be yielded through more granular inquiries—beyond generic surveys as in most existing literature—into students' and instructors' utilization of specific e-learning functionalities and platforms and, where applicable, triangulating usage ("objective" input) with perceptions ("subjective" input). Lastly, we believe that the method employed in our study can be further tailored by other medical institutes for similar studies, because of the large cross-sectional sample which was recruited—by far the largest study examining both students' and instructors' perspectives in medical schools.

## Data Availability

The datasets used and/or analyzed during the current study are available from the corresponding author on reasonable request.
